# Molecular Basis of Cancer Pain Management: An Updated Review

**DOI:** 10.3390/medicina55090584

**Published:** 2019-09-12

**Authors:** Ayappa V. Subramaniam, Ashwaq Hamid Salem Yehya, Chern Ein Oon

**Affiliations:** Institute for Research in Molecular Medicine (INFORMM), Universiti Sains Malaysia (USM), Pulau Pinang 11800, Malaysia; ayappa725@gmail.com (A.V.S.); ashwaqlabwork@gmail.com (A.H.S.Y.)

**Keywords:** cancer, pain management, analgesics, adverse drug effects, polymorphism

## Abstract

Pain can have a significantly negative impact on the quality of life of patients. Therefore, patients may resort to analgesics to relieve the pain. The struggle to manage pain in cancer patients effectively and safely has long been an issue in medicine. Analgesics are the mainstay treatment for pain management as they act through various methods on the peripheral and central pain pathways. However, the variability in the patient genotypes may influence a drug response and adverse drug effects that follow through. This review summarizes the observed effects of analgesics on UDP-glucuronosyl (UGT) 2B7 isoenzyme, cytochrome P450 (CYP) 2D6, μ-opioid receptor μ 1 (OPRM1), efflux transporter P-glycoprotein (P-gp) and ATP-binding cassette B1 ABCB1/multiple drug resistance 1 (MDR1) polymorphisms on the mechanism of action of these drugs in managing pain in cancer. Furthermore, this review article also discusses the responses and adverse effects caused by analgesic drugs in cancer pain management, due to the inter-individual variability in their genomes.

## 1. Introduction

**Figure 1:**
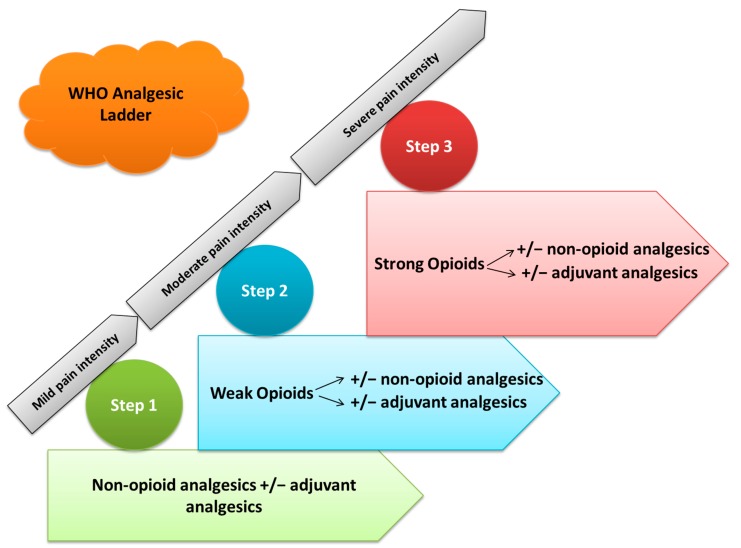
Overview of the analgesic ladder designed by the World Health Organization (WHO).

Pain is often experienced by cancer patients, particularly those in the advanced stage of disease where the prevalence is estimated to be more than 70% [1]. More than three decades ago, the World Health Organization (WHO) designed the 3-step “analgesic ladder” to facilitate and standardize and to advise pharmacologic cancer pain management and advising physicians worldwide on how to improve pain management in their patients (Figure 1) [1].

However, some patients with advanced cancer have inadequate control of pain with systemic analgesics. These patients may alternatively benefit from the invasive techniques such as neuraxial analgesia for vertebral pain, peripheral nerve blocks, sympathetic blocks for abdominal cancer pain and percutaneous cordotomy [2].

Non-opioids, co-analgesics (e.g., nonsteroidal anti-inflammatory drugs), and non-pharmacological measures, are frequently used to enhance analgesic control and lessen opioid requirements. In addition, they are also used to reduce adverse events related to opioid use [3]. A myriad of genes have been studied to identify biomarkers in opioid therapy. These include genes implicated in the pharmacokinetics (*CYP2D6, CYP3A4/5, ABCB1* and *UGTs*,) and pharmacodynamics (*OPRM1* and *COMT*) of opioids [3]. These genes are studied vastly because they play a key role in drug metabolism.

This review focuses on the different types of drugs that are used in cancer pain therapy and the various enzymes, which are involved in the metabolism of these drugs.

## 2. Cancer and Analgesics

According to the World Health Organization (WHO) in 1986, the analgesic ladder is the main reference for cancer pain management [4]. Morphine is used in the third step of the WHO analgesic ladder, which functions to treat moderate to severe pain. This step also consists of additional opioids, (e.g., fentanyl, oxycodone, buprenorphine, hydromorphone and methadone) [5]. Supportive drugs such as laxatives and antiemetics are used alongside the analgesic ladder to prevent adverse effects of opioid treatment [6], as well as non-pharmacological measures (radiotherapy, nerve blockades and neurolytic blocks) [7].

The first step the of analgesic ladder is used for treating mild pain and includes non-opioids analgesics, such as acetaminophen (paracetamol), and non-steroidal anti-inflammatory drugs (NSAIDs) with or without adjuvant analgesics [4,8,9,10]. The second step of the analgesic ladder consists of weak opioids, such as tramadol or codeine, which are used for mild to moderate pain. Lower doses of a step III opioid, such as morphine or oxycodone, should be administered instead of codeine or tramadol, with or without non-opioids analgesics and adjuvant analgesics [5,8,9,10]. The third step of the analgesic ladder treats moderate to strong pain via strong opioids, such as morphine or oxycodone, with or without non-opioids analgesics and adjuvant analgesics [5,8,9,10]. The correct application of the WHO pain ladder can help to successfully manage pain and provide effective analgesia for patients.

Chronic pain remains a disturbingly common consequence of cancer and its treatment. Several studies have found that more than 50% of cancer patients experience moderate to severe pain throughout their lifetime [11]. Opioid analgesia is a mainstay treatment of cancer pain. Opioids have also been associated with cancer recurrence [11,12]. Several studies have demonstrated that opioid drug abusers experience heightened sensitivity to viral and bacterial infections. Furthermore, opioids have been proven to show an effect on the function of the immune system to promote carcinogenesis. Although this is biologically plausible, clinical, in vitro and animal evidence is still inconclusive [13,14]. Recent findings have downplayed this hypothesis by stating that only particular types of tumors that possess particular receptors will be more inclined to react to opioid, either positively or negatively [11]. From these findings, it is suggested that opioids play a pivotal role in the management of moderate to severe cancer pain [11].

## 3. Pain Medication, Opioid Analgesics and Non-Steroidal Anti-Inflammatory Drugs (NSAID) in Cancer Pain Relief

### 3.1. Morphine

Morphine was first isolated from the opium poppy plant by a German pharmacist, Friedrich Sertürner, between 1803 and 1805. It is still one of the most commonly used drugs to achieve analgesia in cancer pain relief. Morphine acts on μ and κ receptors but its analgesic effect is mediated primarily by the μ receptors. This is confirmed by loss of morphine analgesia in μ receptor knockout mice [15]. The main metabolic pathway utilized by morphine is glucuronidation, which produces morphine-6-glucuronide and morphine-3-glucuronide as by-products. Morphine-6- glucuronide possesses a higher analgesic potency than its parent compound [16,17]. These metabolites are removed by the kidneys, therefore, in kidney disease patients’, metabolite concentration may be high and may lead to adverse events [8,18]. A multiple regression analysis, presented maximum pain score as the crucial factor contributing to morphine usage, followed by ethnicity and A118G polymorphism [19]. Advanced cancer patients who suffered pain caused by homozygosity for the 118G allele of the μ-opioid receptor required higher morphine doses to achieve successful pain control. Although the analgesic effects are already partially decreased in heterozygous carriers, the respiratory depressive effects are decreased in homozygous carriers of the variant 118G allele [17]. It has been demonstrated that morphine does not stimulate tumor initiation, however, it does stimulate the growth of an existing breast tumor in a transgenic mouse within an experimental study [20]. Morphine is regarded as an effective analgesic for pain management amongst pediatric patients [9].

### 3.2. Codeine

Codeine is known as a pro-drug: An inactive metabolite that is converted to its active counterpart. Codeine is a weak opioid that is generally administered after surgery and is used alongside certain drugs to manage acute and chronic pain [21]. The analgesic properties of codeine originate from its conversion to morphine and morphine-6-glucoronide by CYP2D6 [3]. Codeine is the parent compound and has a 200× lower affinity at the opioid receptor than its morphine metabolite [22]. Poor metabolizers possess little or no CYP2D6 enzyme activity and may not achieve a sufficient level of pain control, whereas, a person with extra copies of CYP2D6 (ultra-rapid metabolizers) may convert codeine to morphine to a greater extent. However, they may be at increased risk of adverse events such as sedation or respiratory depression [3,16,18]. Codeine usage is not recommended in the presence of renal failure [8,23]. In addition, its administration in pediatric patients has shown low clinical efficacy and limited effect upon dosage escalation [9]. In August 2012, the FDA advised against prescribing codeine for children after tonsillectomy due to the risk of CYP2D6 life threatening overdose attributed to genetic variation [24].

### 3.3. Hydrocodone

Hydrocodone is often used in patients with advanced cancer. It is metabolized by CYP2D6 to form the active metabolite hydromorphone, which has a 10× to 33× greater affinity for μ-opioid receptors than hydrocodone [3]. A study has demonstrated an effect of CYP2D6 polymorphisms on hydrocodone metabolite production [25]. Although the effect of CYP2D6 polymorphisms on metabolite production was reported in pharmacokinetic studies, the pain relief experienced by a child who is given hydrocodone is not dependent on metabolism by CYP2D6 [22]. Adverse effects of hydrocodone are similar to other opioids [8]. Hydrocodone is a viable option for children that are known to have a poor metabolizing phenotype [22].

### 3.4. Hydromorphone

Hydromorphone has a similar structure to morphine and is available as parenteral and oral products [8]. Its potency and high solubility may be beneficial for patients who require a high opioid dosage and for subcutaneous administration [8,18].

### 3.5. Fentanyl

- Fentanyl is a highly lipophilic opioid and is used for relieving cancer pain in transdermal and transmucosal immediate-release formulations [18]. It is metabolized primarily by CYP3A4/5 to the inactive metabolite, norfentanyl [3]. A118G polymorphisms of *OPRM1* were present in various Asian cohorts’ post-surgery and revealed lower fentanyl requirements in A118G-homozygous individuals [26]. The use of fentanyl for children above the age of 2 years has been approved by the FDA and it is one of the most commonly used analgesics amongst pediatric patients [9]. Comparisons made between morphine and transdermal fentanyl have shown an equal analgesic efficacy [8]. Fentanyl can be administered by continuous intravenous or subcutaneous infusion [18]. All the studies found transdermal fentanyl to be cost-effective against oral sustained release morphine with incremental cost-effectiveness ratios of £17,798, £14,487 and £1406 per quality adjusted life years in the studies by Neighbors et al. [27], Radbruch et al. [28] and Greiner et al. [29], respectively for cancer and non-cancer patients with moderate to severe chronic pain [30]. In one study of 60 adult patients with cancer receiving transdermal fentanyl, showed that polymorphisms in the gene *ABCB1* could lead to significant changes in fentanyl plasma concentrations, with the ABCB1 1236TT variant being associated with a lower need for rescue medication. To date there have been no statistically significant findings for fentanyl-related adverse effects, in the previous study or current body of literature [31,32].

### 3.6. Sufentanyl

Sufentanyl is often used as a replacement to fentanyl when the volume of fentanyl needed for treatment is above the range which can be administered through an injection [33,34,35]. Sufentanyl is more effective at a lower dose for pain control among patients [33]. It is mostly used for the treatment of patients with renal impairment [34].

### 3.7. Methadone

The pharmacokinetics of methadone is highly variable. Methadone is a synthetic opioid, which is commonly used as a second-line option in the presence of neuropathic pain in cancer and recognized for its use in the treatment of opioid dependency [26]. Its average half-life is approximately 24 h and can range from less than 15 hours to more than 130 hours. Results from an elegant study by Mercadante and colleagues reported that methadone achieved an analgesic effect and was more stable than morphine in a sample of 40 patients who were treated for two or three times daily according to their clinical needs [8,36]. Its increased usage has become associated with a high rate of serious adverse effects, particularly in populations with non-cancer related pain. However, methadone has a complex metabolism that involves both CYP3A4 and CYP2D6 and is a weak inhibitor of serotonin reuptake [18].

### 3.8. Levorphanol

Levorphanol is a potent opioid and is similar to methadone and morphine. Levorphanol has a strong affinity for μ, κ and δ opioid receptors [8]. Studies have indicated that levorphanol is an effective treatment for chronic neuropathic pain [8].

### 3.9. Buprenorphine

Buprenorphine has a high affinity for the μ receptor. Transdermal buprenorphine is available at a higher dosage formulation in other countries compared to the United States and is used for managing cancer pain [18]. Buprenorphine is converted to an active metabolite called norbuprenorphine by CYP3A4 and CYP2C8 metabolism, which is a weaker but full-opioid agonist [8]. Liver disease can affect the metabolism of buprenorphine [8]. Administration of buprenorphine to opioid naive patients or those receiving low-dosage opioid regimen, may induce withdrawal symptoms if physical dependence is present. Therefore, it is wise to limit treatment to cancer patients [18]. In addition, Greiner et al. [29] showed transdermal buprenorphine to be cost-effective against oral sustained release morphine with an incremental cost–effectiveness ratio of £6248 per quality adjusted to years of survival [29,30].

### 3.10. Oxycodone

Oxycodone is an oral opioid treatment choice for chronic cancer pain, and it is a semi-synthetic opioid prescribed for moderate to severe pain [25]. Oxycodone binds to both μ and κ receptors, but there is uncertainty surrounding the clinical implications of this dual binding [18]. Like hydrocodone, the parent compound possesses identical levels of activity at the opioid receptor as the metabolites [22]. CYP3A4 metabolizes most of the oxycodone to noroxycodone. A smaller percentage is metabolized by CYP2D6 to the active metabolite, oxymorphone, which has a 40× higher affinity and 8× higher potency for μ-opioid receptors than oxycodone [3]. The response of poor metabolizers was from 2× to 20× less than those individuals who were extensive metabolizers [26,37]. Catalyzed by CYP3A4 and CYP2D6, oxycodone is undergoing metabolism in the liver through four different metabolic pathways. When compared to morphine, the resulting metabolites, had different affinities for the μ-opioid receptor, from highest to lowest: oxymorphone > morphine > noroxymorphone > oxycodone > noroxycodone [26]. Oxycodone was more effective than other strong opioids at decreasing pain intensity scores and resulted in a lower incidence of nausea and constipation, suggesting that this drug offers better pain management among cancer patients [10]. Oxycodone was reported to be effective in patients with pancreatic cancer and this was indicated by a significant drop in pain score within 4 weeks [10,38]. The ultra-rapid metabolizer group experienced side effects such as sedation and reduced oxygen saturation more frequently compared to the poor metabolizer group [37]. Patients who are administered oxycodone often experience opioid-induced constipation (OIC) as a side effect of the treatment. In order to counter this, naloxone is co-administered to alleviate or reduce the occurrence of OIC [39]. Naloxone works in terms of binding to the μ-receptor in the gastrointestinal tract. A clinical study comprising 128 patients has revealed that there is no difference in analgesic efficacy between the control group, oxycodone only, and the oxycodone/naloxone treated group [40].

### 3.11. Tramadol

Tramadol is recommended for patients with moderate, severe nociceptive or neuropathic pain. It is used widely in certain countries, particularly amongst cancer patients who are opioid naive or have limited opioid exposure [18]. It consists of two enantiomers and is a synthetic analog of codeine and morphine, both of which promote analgesic activity via different mechanisms [26]. Tramadol undergoes CYP2D6 dependent O-methylation to demethyltramadol (M1) [26]. However, M1 however has a much higher affinity for the μ-receptor compared to the parent compound but a lower affinity compared to strong opioids [8,17,26]. A higher tramadol dosage will be required to achieve satisfactory pain relief in CYP2D6 PMs compared with Ems [3,41]. (−)-Tramadol inhibits norepinephrine reuptake and (+)-tramadol inhibits serotonin reuptake, thus, pain transmission in the spinal cord is greatly inhibited [26]. Poor metabolizers are characterized by deficient O-demethylation and displays two inactive alleles, resulting in their inability to convert tramadol to O-demethyltramadol and as a consequence, inadequate analgesia [17]. A case report documented that a pediatric ultra-rapid metabolizer experienced respiratory depression following tramadol administration, despite tramadol being a partial opioid [25,26,42]. It has been reported that tramadol is not commonly used to manage pain in pediatric patients and very little data exists for young patients below 16 years of age [9]. Examples of adverse effects produced by tramadol include: Constipation, dizziness, nausea, sedation, dry mouth and vomiting [8].

### 3.12. Tapentadol

Tapentadol is structurally similar to tramadol and is approved for use in the treatment of severe chronic pain in cancer patients [8]. Tapentadol binds with high affinity to μ, κ and δ opioid receptors. It acts on the µ-opioid receptor and inhibits noradrenaline reuptake [43]. Tapentadol provides analgesic efficacy similar to that of oxycodone when it is first administered at low doses in opioid-naïve patients [43]. However, the incidence of gastrointestinal adverse effects has been reported to be lower in the tapentadol group than in the oxycodone group [43]. Limited occurrence of gastrointestinal adverse side effects from tapentadol may serve as a great advantage in pain management in the context of multifactorial diseases, such as cancer, where other drugs can contribute to induce nausea, vomiting or constipation. However, tapentadol is a relatively new drug and there is minimal published information on its use in cancer pain management [18].

### 3.13. Non-Steroidal Anti-Inflammatory Drugs (NSAID)

NSAIDs are a group of non-opioid analgesics, which are commonly used for the treatment of acute pain, following surgery or chronic pain [21]. NSAIDs are used alone or alongside opioids, which treat moderate to severe pain. Many NSAIDs are metabolized by the cytochrome enzyme CYP2C9 [44]. Poor metabolizers possess lower CYP2C9 activity compared to wild-type. This results in increased area under the plasma drug concentration–time curve, decreased NSAID clearance and feasibly an increased risk of adverse effects [42]. For multiple NSAIDs, which include flurbiprofen, piroxicam, R-ibuprofen, tenoxicam and celecoxib (a COX-2 inhibitor), the CYP2C9 genotype is an important indicator of metabolic clearance. Individuals who possess the wild-type CYP2C9*1 genotype have a significantly lower systemic exposure compared with individuals that have possessed the CYP2C9*3 genotype [41,45]. Variations in CYP2C9 and CYP2C8 impair the clearance of ibuprofen from the body. This means that the medication remains in the body for much longer than it should, potentially leading to adverse effects, such as gastrointestinal bleeding [46]. Hussain and colleagues reported that NSAIDs have a strong, potential anti-cancer effect. Inhibition of PGE2 production (in addition to COX-2 inhibition) may play a vital role in cancer cell mutation and proliferation. Ultimately, inhibition of PGE2 could possibly stimulate cell mediated immune response, in so doing increasing the cytotoxic abilities of NK cells [20,47].

### 3.14. Paracetamol (Acetaminophen)

Paracetamol (N-(4-hydroxyphenyl)-acetamide) is one of the most widely used over-the-counter analgesics [48]. Paracetamol has been frequently co-administered with analgesics for treating cancer patients [8,10,18,26,49]. Paracetamol is regarded as the drug of choice for children with pain of a non-inflammatory nature [9]. Glucuronidation was initially recognized to be impaired in patients with Gilbert’s syndrome, which is an inherited bilirubin disglucuronidation condition that increases the risk of paracetamol toxicity in affected individuals. A toxic intermediary metabolite, *N*-acetyl-p-benzoquinoneimine (NAPQI) is produced from cytochrome P450 2E1 and 3A4 metabolism of paracetamol [48]. NAPQI is a toxic compound. An overdose of paracetamol may cause a build-up of NAPQI, leading to paracetamol-induced acute liver failure. A systematic review carried out by Wiffen and colleagues, have reported that there is no clear evidence that paracetamol used alone or in combination with opioid was able to provide significant pain relief to cancer patients [50].

### 3.15. Nefopam

Nefopam is a common postoperative non-opioid, non-steroidal analgesia. The main mechanism of analgesic action of nefopam is through the inhibition of serotonin, norepinephrine and dopamine reuptake [51,52,53,54,55]. A clinical study conducted by Kim and colleagues have demonstrated that patients who were administrated nefopam 48 hours post renal transplant operation consumed 19% less fentanyl compared to the control group. This study suggests that nefopam as an adjunct to standard analgesia, fentanyl, reduced postoperative fentanyl consumption besides also providing better analgesia [54]. In a novel review by Girard et al. [55], they have compiled the studies where nefopam was used in combinations with opioids, paracetamol and non-steroidal anti-inflammatory drugs in both preclinical and clinical setting. The results have shown that nefopam used in combination with all these drugs has a significantly better analgesic effect in both settings [55]. Nefopam has also shown to reduce acute and chronic postoperative breast cancer surgery for a study group involving 41 patients, where the patients were administered preventive nefopam. In addition, nefopam has also been reported to reduce chronic pain [56]. Another study conducted by Hwang and colleagues have demonstrated that nefopam used in combination with oxycodone reduced the incidence of nausea among 60 patients 6 h post gynecologic surgery [57].

### 3.16. Metamizole (Dipyrone)

Metamizole is a widely used non-opioid analgesics for the treatment of cancer pain however it is banned in several countries due to its toxicity towards patients who have agranulocytosis [58,59]. Gaertner and colleagues reported a systematic review, which highlighted that metamizole used alone or in combination with opioid were effective in reducing cancer pain. They also reported that at higher doses, metamizole was as effective as morphine 60 mg/day [58,60]. Metamizole in combination with magnesium chloride was shown to reduce cancer pain while also preventing tolerance in a study conducted with murine model of cancer [61]. Hearn and colleagues detailed in their systematic review, from eight studies, 70% of the adult patients with acute postoperative pain who were treated with a single dose of metamizole experienced at least 50% of maximum pain relief over 4–6 h [59].

## 4. Adjuvant Analgesics in Cancer

**Figure 2:**
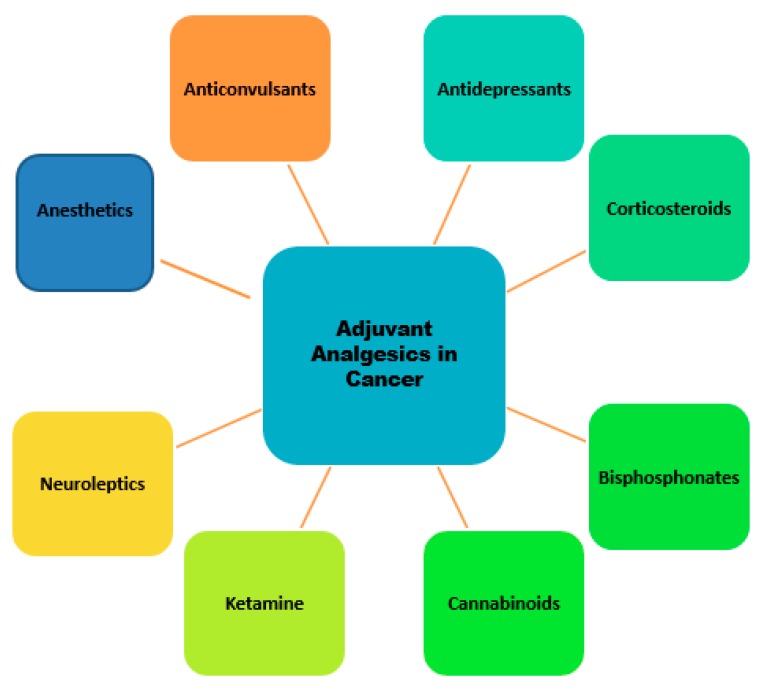
Schematic diagram of adjuvant analgesic drug categories for cancer pain therapy.

Adjuvant analgesic refers to drugs that are marketed for indications other than pain but with analgesic properties in some painful conditions [49]. Drugs such as opioids, non-steroidal anti-inflammatory drugs (NSAIDS), and acetaminophen are usually co-administered with analgesics when treating cancer pain, although they can be used alone. Adjuvant analgesics are usually added to an opioid to reduce adverse effects and to enhance pain relief from opioid [49]. Over the past three decades, the use of these drugs used in clinic has increased dramatically and several are administered as first-line drugs in the treatment of chronic non-malignant pain. However, in cancer pain management, conventional practice has evolved to view opioids as first-line drugs and adjuvant analgesics are usually considered after opioid therapy has been optimized [49]. Adjuvant analgesics are specific for neuropathic pain, which was most recently defined by the International Association for the Study of Pain (IASP) as “Pain arising as a direct consequence of a lesion or disease affecting the somatosensory system“ [13,62]. Of cancer pain 40%–50% was characterized by surveys to be wholly or partially neuropathic [49]. The adjuvant analgesics consist of classes of medications with different primary indications (Figure 2). According to conventional use, a group of non-specific analgesics can be differentiated from those used for specific indications, including bone and neuropathic pain.

### 4.1. Antidepressants

Antidepressants are a mainstay treatment for neuropathic pain. Antidepressants are most commonly used to treat patients with a history of depression cases. Tricyclic antidepressants (TCA) and the selective serotonin reuptake inhibitors (SSRIs) are common antidepressants used in pain management among patients [8,63]. Serotonin norepinephrine reuptake inhibitors (SNRIs) is a new class of antidepressant used for the treatment of neuropathic pain and is more effective than SSRIs [63]. Antidepressant drugs used in combination with opioid has shown an opioid-sparing effect [63]. TCAs are not recommended to be used for elderly and heart disease patients due to frequent adverse side effects [8,49]. TCA could also potentially exacerbate hypotension among the elderly patients [8]. There are limited studies conducted on antidepressant effects on cancer pain now, as such, future studies should explore this area to provide better pain management among cancer patients.

### 4.2. Corticosteroids

Corticosteroids are usually used for cancer pain management. Studies have shown that corticosteroid treatment improves the patient’s appetite and reduces nausea [49,63]. Bone pain associated metastasis are commonly treated by corticosteroids [63]. Patients who are treated by this treatment may experience sides effects such as hypertension, hyperglycemia, osteoporosis and immunosuppression [63]. High doses of corticosteroids treatment are administered for patients who experience acute pain or spinal cord compression [49,63].

### 4.3. Anticonvulsants (Gabapentinoids)

The most common anticonvulsant drugs used for managing cancer induced neuropathic pain are gabapentin and pregabalin [8]. Gabapentin must be dose adjusted to avoid the occurrence of adverse events [8,49]. Gabapentin has been shown to reduce cancer induced bone pain, which is caused by bone metastasis besides also, reducing postoperative bone pain [64,65,66]. Pregabalin is structurally similar to gabapentin but is more potent than its predecessor [63]. Both gabapentin and pregabalin were reported to provide effective pain relief post breast surgery [67,68,69,70]. Gabapentinoids are excreted through the renal route, hence patients with renal failure will require lower doses [13]. Phenytoin is another drug in this category that can be used to treat cancer pain [49]. Other anticonvulsant agents have not been studied extensively with regards to cancer pain management [8]. A novel study by Bugan et al. [71] has reported that gabapentin causes pro- and antimetastatic effect.

### 4.4. Anesthetics

Lidocaine may be administered through the oral, subcutaneous, parenteral and transdermal route [8]. Cancer pain was significantly reduced in a study conducted by Sharma and his colleagues [8,72]. Lidocaine provided prolonged pain relief. Lidocaine are more commonly used for non-neuropathic pains [49]. Lidocaine can be used in combination with other anticonvulsant drugs for patients who response positively to intravenous lidocaine treatment [63]. Another study has reported that the application of topical lidocaine before the surgery has significantly reduced post-surgery pain for breast cancer patients [63,73].

### 4.5. Ketamine

The use of ketamine in the management of cancer related neuropathic pain produced opioid-sparing effect [8,49,74]. Patients under hospice care are usually administered ketamine on a long-term basis until they pass on [49]. In a contradictory a review written by Jonkman et al. [75], they summarized from four controlled trials that there is lack of evidence that ketamine provides opioid-sparing effect for cancer pain. However, they have also argued that the efficacy of ketamine as a treatment for cancer pain management was not completely ruled out [74,75,76,77,78].

### 4.6. Neuroleptics

Patients administered with olanzapine was shown to have reduced pain scores and improved cognitive function besides also reduced anxiety [8]. Consumption of opioid after administration of neuroleptics was reported to be decreased [49].

### 4.7. Bisphosphonates

Bisphosphonates are used in adjunction during treatment of cancer due to the high occurrence of cancer induced bone pain. Patients under palliative care often experience bone pain. Bisphosphonates have been shown to improve pain management among patients with breast, prostate or lung cancer [8]. One bisphosphonate that has been studied widely is pamidronate, which has shown its efficacy in breast cancer patients [49]. The bone density of patients treated with pamidronate improved over time [63]. Another drug, zoledronic acid, which is more potent compared to pamidronate, also decreased cancer induced bone pain in breast, lung, myeloma and prostate cancer [49].

### 4.8. Cannabinoids

There are limited studies on cannabinoids use in cancer pain management as of now. Tetrahydrocannabinol (THC) and cannabidiol are the two most abundant compounds found in the cannabis plant. Some studies have suggested that the use of cannabinoids as adjunct to opioid, provided significant pain relief [64,79]. Cannabinoids efficacy in treatment of cancer pain may vary based on the population race [80]. Appetite of patients who are under cannabinoid treatment are improved [79,81]. Based on a summarized table produced by Bennett et al. [82], it was clear that the use of cannabinoids for the treatment of neuropathic and cancer related pain, decreased pain with mild adverse effects. The combination of THC and cannabidiol as a treatment has shown to provide significant pain relief among patients [81]. In a recent systematic review by Tateo [83], from eight low or moderate quality randomized clinical trials, cannabinoids were reported to effectively manage cancer pain when administered in combination with opioid. Nonetheless, further investigations must be carried out on the effectiveness of cannabinoids as adjuvant analgesics. There is no clear evidence that cannabinoids are beneficial for the treatment of cancer pain [80,83,84,85].

### 4.9. Dexmedetomidine

Dexmedetomidine is sedative drug that is usually used in the intensive care units or for patients who are under hospice or palliative care. Based on a case study reported by Hilliard and colleagues [86], the drug managed to clear the patient’s pain and delirium towards the end of her life while also allowing the patient to maintain wakefulness and interact with family members. Two other case studies also revealed that administration of dexmedetomidine, provides opioid-sparing effect and essential for end-of-life care [87,88]. In an elegant study by Yuan and colleagues [89], they have proven that the combination treatment of dexmedetomidine with tramadol provided a better analgesic effect compared to the high dose treatment of tramadol alone in bone cancer rat models. Dexmedetomidine used as an adjunct along with bupivacaine for a single-shot paravertebral block was shown to improve analgesia lasting duration post breast cancer surgery. The combination also reduced opioid consumption and the nausea episodes among patients [90].

## 5. Enzymes Involved in Drug Metabolism

### 5.1. CYP2D6

One of the most common CYPs involved in drug metabolism is cytochrome P450 family 2, subfamily D, polypeptide 6 (CYP2D6). In this enzyme in which the metabolic rate can fluctuate by over 100× between the allelic variants expressed in different ethnic groups [21,91]. The genetic polymorphism of this enzyme may result in the generation of four different phenotypes. These are poor metabolizers, intermediate metabolizers, extensive metabolizers and ultra-rapid metabolizers. An individual with a genotype of two non-functioning alleles is a poor metabolizer (PM); at least one reduced functioning allele is an intermediate metabolizer (IM); at least one functional allele is an extensive metabolizer (EM) and multiple copies of a functional allele is an ultra-rapid metabolizer (UM). EM is the most common phenotype [22]. It has been shown that opioids have adverse events in patients at both extremes of function, ultra-rapid and poor. It is for this reason, that we consider both metabolizer extremes (ultra-rapid and poor) as dysfunctional and recommend that CYP2D6 substrate drugs and prodrugs be avoided in these patients [92]. In one non-lethal case, a cancer patient with pneumonia given codeine for cough suppression went into respiratory arrest. Genotyping characterized the patient as a CYP2D6 ultra-rapid metabolizer with a functional gene expansion. Death was averted when the patient was treated with naloxone and fully recovered [92,93]. Analysis on the genetic makeup of patients is crucial in determining the effectiveness and safety of the treatment to avoid adverse events or death (Table 1). The distribution of the CYP2D6 phenotypes varies by ethnicity, mainly due to differences in inherited SNPs [42]. Therefore, determining the status of CYP2D6, could provide guidance in giving out prescriptions and optimize overall cost effectiveness of health care services [24].

### 5.2. CYP2C9

CYP2C9 is the most abundant P450 cytochrome in the liver. Almost 15% of clinically useful drugs, including various NSAIDs is metabolized by this enzyme in the first phase of drug metabolism [48]. Phase 1 metabolism of xenobiotic compounds is important to introduce functional groups or polar groups into the compounds. The products of phase 1 metabolism will readily couple with an endogenous conjugating molecule, which makes the metabolite less toxic and easily eliminated from the body [112]. Over 50 variants have been identified from the highly polymorphic gene that codes for CYP2C9. CYP2C9 polymorphisms may play a significant role in NSAID toxicity. Although many non-steroidal anti-inflammatory drugs (NSAIDs) are metabolized by CYP2C9, such as suprofen, naproxen, diclofenac, ibuprofen, ketoprofen, meloxicam, piroxicam, flurbiprofen, indometacin and tenoxicam. There is a difference in the effectiveness of metabolic clearance between the different NSAIDs [41]. CYP2C9 activity in poor metabolizers are lower compared to the wild type [42].

### 5.3. Opioid Receptors

Opioid receptors belong to a family of G-protein-coupled receptors (GPCRs), which are located in the brain and spinal cord [25,48]. There are three types of classical opioid receptors: mu (μ), kappa (κ) and delta (δ). The μ-opioid receptor (OPRM1) is the main binding site for various opioid drugs and beta-endorphins [21]. A common polymorphism of OPRM1 is a single nucleotide substitution at position 118, where an adenine is substituted for a guanine (A118G). It was reported that among Caucasians these substitutions occur with an allelic frequency of 10%–30%, with a higher prevalence amongst Asians, and a lower in African Americans [19]. The binding affinity for b-endorphin is increased with this polymorphism, which results in the change of an amino acid (asparagine for aspartate). This affects the action of opioids at the receptor [37]. Adverse effects of the drugs, such as vomiting, pupil dilation, nausea and sedation, are reduced in association with the G allele. Therefore, carriers of the G118 allele may accept higher opiate doses than non-carriers [44,91]. 118A homozygotes or heterozygotes consumed considerably less morphine than patients with 118G homozygotes [91]. Cancer patients with an 118GG polymorphism in the *OPRM1* gene need a higher morphine dose than patients with 118AA (1,2,3). Other genes, such as *CREB1*, *GIRK2* and *CACNA1E*, have similar consequences on the pain-relieving effects of opioids. Genotypes related to morphine’s ability to treat pain, such as the GG genotype for *OPRM1*, may help inform appropriate dose selection. In one study, patients with the GG genotype often require higher daily doses of morphine to achieve appropriate levels of analgesia, in comparison to the wild-type A allele (225 + 143 mg/day vs. 97 + 89 mg/day in those with the A allele for *OPRM1*, *p* = 0.006) [31,32,93]. More than 100 variants of the receptor gene (*OPRM1*) have been identified [113].

### 5.4. Adenosine Triphosphate-Binding Cassette, Sub-Family B, Member 1 (ABCB1)

Adenosine triphosphate (ATP)-binding cassette subfamily B or multi-drug resistance gene (*MDR1*) encodes for P-glycoprotein [48]. P-glycoprotein is an efflux transporter that actively pumps substrates out of tissues to decrease concentrations of drugs on the body [113]. These proteins are present in a variety of human tissues, including the kidney, liver, gastrointestinal tract and brain [42]. Decreased renal excretion, increased bioavailability of oral medications, or in central nervous system concentrations are result of damaged the P-glycoprotein transporters. Specifically, variations in ABCB1 transporters in the brain may affect the transport of opioids into the brain through the blood–brain barrier [3].

### 5.5. Catechol-O-Methyltransferase (COMT)

The catechol-O-methyltransferase (COMT) enzyme is one of the enzymes that metabolizes the catecholamines, norepinephrine, epinephrine and dopamine. Therefore, the COMT enzyme acts as the main modulator of dopaminergic and adrenergic/noradrenergic neurotransmission [15]. Patients who are treated for cancer-related pain may experience opioid-related side effects if they possess a genetic variation in COMT [48]. Improved dopaminergic transmission was reported in Val158 allele, which exhibits a high COMT activity, which has been suggested to confer an advantage in the processing of aversive stimuli or in stressful conditions. In contrast, advantage in memory and attention tasks may be associated with the Met158 allele [19]. The Met158 variant is the most widely studied variant, where a G to A nucleotide substitution at codon 158 may produce an amino acid change from valine to methionine, resulting in individuals who have homozygous methionine-158 genotype [42,91]. Cancer patients with the Met/Met genotype have demonstrated a lower need for morphine compared with those with a Val/Val genotype [91]. The effect of polymorphisms in the OPRM1 and COMT genes, which transcribe opioid receptor μ 1 and catechol-O-methyltransferase respectively, are relatively well categorized in their effect on acute postoperative, cancer-related and chronic pain. When patients are homozygous for the common amino acid substitution val158met, they require a dose of morphine that is significantly higher than homozygous met/met patients [31,32,93].

### 5.6. Uridine Diphosphate Glucuronosyltransferases (UGTs)

The uridine diphosphate glucuronosyltransferases (UGTs) family serves a major role in the conjugation of potentially toxic drugs and endogenous compounds. UGTs catalyze the glucuronidation reaction, resulting in the addition of glucuronic acid to several lipophilic compounds [15]. Although abundant in the liver, UGTs are also found throughout different parts of the body, including the kidneys, colon, prostate, stomach and small intestines [113]. Uridine glucuronyl transferase (UGT) enzymes are subdivided into four families, and each of these into subfamilies [16]. Morphine is primarily metabolized by UDP-Glucuronosyltransferase-2B7 (UGT2B7), a phase 2 isoenzyme. It is metabolized in the liver into two metabolites: Morphine-3-glucuronide (M3G) and morphine-6-glucuronide (M6G) [42]. UGT2B7 is linked to altered levels of mRNA expression and enzymatic activity with different metabolite production [48]. The polymorphism in UGT2B7 may lead to different rates of morphine glucuronidation resulting in higher or lower levels of morphine/metabolite ratios [17,42]. Genotypic differences in UGT2B7, which is responsible for metabolizing morphine into morphine-6-glucuronide and morphine-3-glucuronide, can impact codeine’s therapeutic effect. In particular, the UGT2B7*2/*2 genotype, which results in a reduced function of the enzyme, has been associated with higher toxicity. Several pharmacokinetic studies have illustrated the effects of these phenotypes on metabolite formation. In one study, a single dose of 30 mg codeine was administered to 12 UM individuals in comparison to 11 EMs and three PMs. Significant differences were detected between EM and UM groups for areas under the plasma concentration versus time curves (AUCs) for morphine with a median (range) AUC of 11 (5–17) μg·h·L^−1^ in EMs and 16 (10–24) μg·h·L^−1^ in UMs relative to individuals with the PM phenotype (0.5 μg·h·L^−1^, *p* = 0.02) [31,32].

### 5.7. Melanocortin-1 Receptor Gene

Variation in the *MC1R* gene indicated the potential for highly targeted analgesia on gender and other differences. There is evidence that women, respond to κ-induced analgesia more than men [91]. Women with either one or no MC1R variants, or to men with two inactivating MC1R variants was reported to experience a weaker analgesic effect from pentazocine (k-opioid agonist) compared to women with two non-functional MC1R alleles [37,41]. Women with redhead and pale skin phenotypes have been shown to have this MC1R gene variation [41,91].

## 6. Conclusions and Future Perspective

Pain management regimes are established to care for post-operative and palliative patients to improve their quality of life. Patients suffering from cancer are usually subjected to chemotherapy treatment, which can be painful and cause uneasiness. Therefore, patients will resort to analgesics. The human genome is highly complex and consists of various types of polymorphisms that differ from one individual to another. A plethora of drugs are discovered and introduced into the market to counter this issue because there is not a specific drug that is suitable for every patient. Administering an incorrect drug to a patient can be life threatening. Hence, it is crucial to have the correct information on individual patient pharmacogenomics at the time of a care decision so that the data can be used to guide therapeutic decision-making. Pharmacogenomics can be employed as the future of analgesic administration to investigate the drug metabolizing enzymes or disease genes, RNA expression or protein translation of genes affecting drug response, inter-individual genetic variability in DNA sequence of drug targets and drug safety [25]. Serious adverse drug reactions (ADRs) or unsuccessful therapeutic effect in some patients may still occur from a medication with proven efficacy and safety [21]. The variation in the reaction to the drugs is often caused by the genetic composition of the individual, which could possibly be an inherited variance or an acquired variance due to mutations in their DNA. Each patient’s genetic coding may be used as a basis for an individualized pain management treatment plan for analgesic metabolism and pain sensitivity allowing efficient and accurate treatments for patients [114]. Opioids like morphine, codeine and tramadol [3] are potent analgesics and serve as the foundation for severe pain management in cancer. The pursuit of personalized medicine has always been the main objective for both physicians and pharmaceutical industries. The main objective in the era of personalized medicine is to administer the correct drug at the precise dose for the “right patient” as the human genome becomes easily accessible [21,42].

## 7. Limitations

The limitation to this review is that the data and information collected are from low or moderate quality articles. In addition, more studies need to be carried out to fully understand the interactions of cancer drugs and painkillers that may affect therapeutic outcome. Hence, this article should only serve as a baseline and reference for future research to be carried out.

## Figures and Tables

**Table 1:** Genetic variants analyzed on the effectiveness and safety of the administered treatment.

Analgesics	Study Type	Genetic Variants	Side Effect	References
Morphine	Non-randomized clinical trial	Multidrug resistance-1 gene (*MDR-1*)	Moderate or severe drowsiness and confusion or hallucinations.	[94]
		Catechol-O-methyltransferase (COMT) enzyme		
		Single nucleotide polymorphisms (SNPs) in intron 1		
	In vitro study- breast cancer cell lines	*NET1* gene expression (mediating the direct effect of morphine on breast cancer cell migration)		[95]
Codeine	Non-randomized clinical trial	*CYP2D6* gene	Sedation, addiction, dizziness and constipation	[96]
Hydrocodone	Observational study	*CYP2D6* gene	Dizziness and constipation	[97,98]
Hydromorphone	Non-randomized clinical trial	*CYP2D6* gene	Dizziness and constipation	[97,99]
Fentanyl	Non-randomized clinical trial	*CYP3A5* and *ABCB1* gene polymorphisms	Dry mouth, wheal and flare	[100]
	Observational study	Genetic variants rs12948783 (*RHBDF2*) and rs7016778 (*OPRK1*)		[101]
Methadone	Randomized double-blind study	*ABCB1*, *OPRM1* gene polymorphisms	Constipation, nausea, dizziness and delirium	[102,103]
Levorphanol	Non-randomized clinical trial	*-*	Nausea and vomiting	[104]
Buprenorphine	Non-randomized clinical trial	Polymorphisms in *OPRD1*	Dizziness, dry mouth, thirst and nausea	[105,106]
Oxycodone	Non-randomized clinical trial	*CYP3A5*	Nausea, vomiting, constipation, lightheadedness, dizziness or drowsiness	[107]
Tramadol	Randomized double-blind placebo controlled cross over study	*CYP2D6*	Dizziness, headache, drowsiness, nausea, vomiting, constipation, lack of energy, sweating and dry mouth	[108]
Tapentadol	Non-randomized clinical trial	No genetic variation	Nausea, vomiting, constipation, fatigue, dizziness, sleepiness, drowsiness and dry mouth	[109]
Paracetamol (Acetaminophen)	Randomized double-blind placebo controlled parallel group study	*COX-3*	Low fever with nausea, stomach pain and loss of appetite	[50]
Non-steroidal Anti-Inflammatory Drugs (NSAID)	Randomized, double-blind, placebo-controlled	*COX-1/COX-2*	Stomach pain, heartburn, stomach ulcers, a tendency to bleed, headaches, dizziness and ringing in the ears	[110,111]
